# Supplementary material for: SDG26 Is Involved in Trichome Control in Arabidopsis thaliana: Affecting Phytohormones and Adjusting Accumulation of H3K27me3 on Genes Related to Trichome Growth and Development
Source: Plants (Basel). 2023 Apr 14;12(8):1651. doi: 10.3390/plants12081651 (PMC10143075; doi:10.3390/plants12081651)
Supplement: Supplementary file 1 [file plants-12-01651-s001.zip › plants-2342896-supplementary.pdf]

Supporting Information

SDG26 Is Involved in Trichome Control in *Arabidopsis thaliana*: Affecting Phytohormones and Adjusting Accumulation of H3K27me3 on Genes Related to Trichome Growth and Development

Jing Zeng [1,2], Lanpeng Yang [3], Minyu Tian [1,2], Xiang Xie [2], Chunlin Liu [1,2,*] and Ying Ruan [1,2,*]

[1] Key Laboratory of Hunan Provincial on Crop Epigenetic Regulation and Development, Hunan Agricultural University, Changsha 410128, China
[2] Key Laboratory of Crop Physiology and Molecular Biology of Ministry of Education, Hunan Agricultural University, Changsha 410128, China
[3] School of Energy and Environment and State Key Laboratory of Marine Pollution, City University of Hong Kong, Kowloon, Hong Kong 999077, China
* Correspondence: liucl@hunau.edu.cn (C.L.); yingruan@hotmail.com (Y.R.)

Content:

Supplementary Figures, Figures S1–S4

Supplementary Tables, Table S1

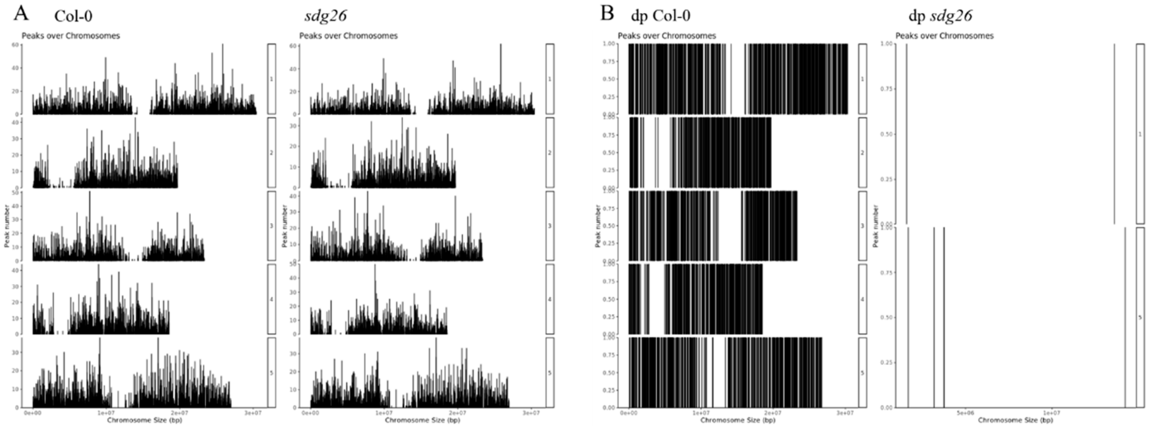

Figure S1. Distribution of enrichment regions of histone modification in chromosomes of Col-0 and *sdg26*. A. Distribution of all detected peaks in chromosomes of Col-0 and *sdg26*. The statistical method is to calculate how many peaks cover each based on the chromosomes. B. Distribution of differential peaks in chromosomes of Col-0 and *sdg26*. The statistical method is to calculate how many peaks cover each based on the chromosomes, with a difference multiple of 3 times.

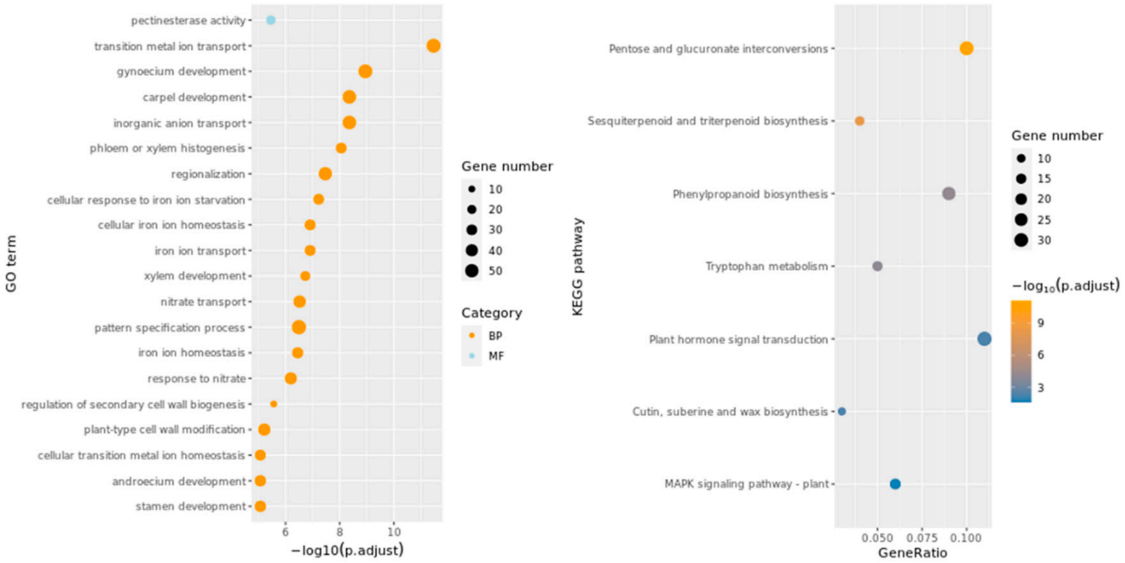

Figure S2. GO and KEGG pathway enrichment analysis of differentially expressed genes associated with protein modification.

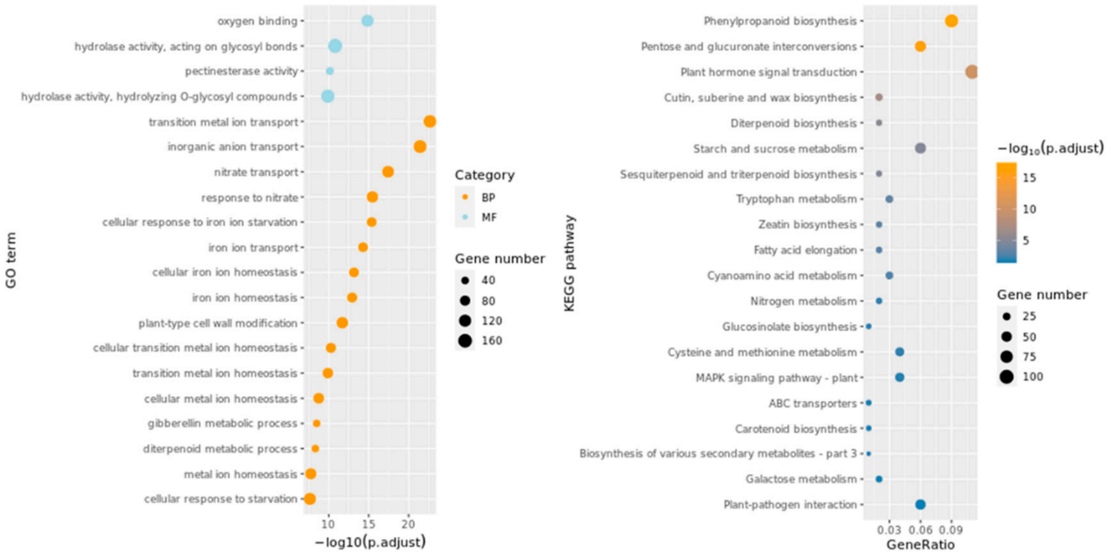

Figure S3. GO and KEGG pathway enrichment analysis of genes associated with histone modification enriched regions in sdg26.

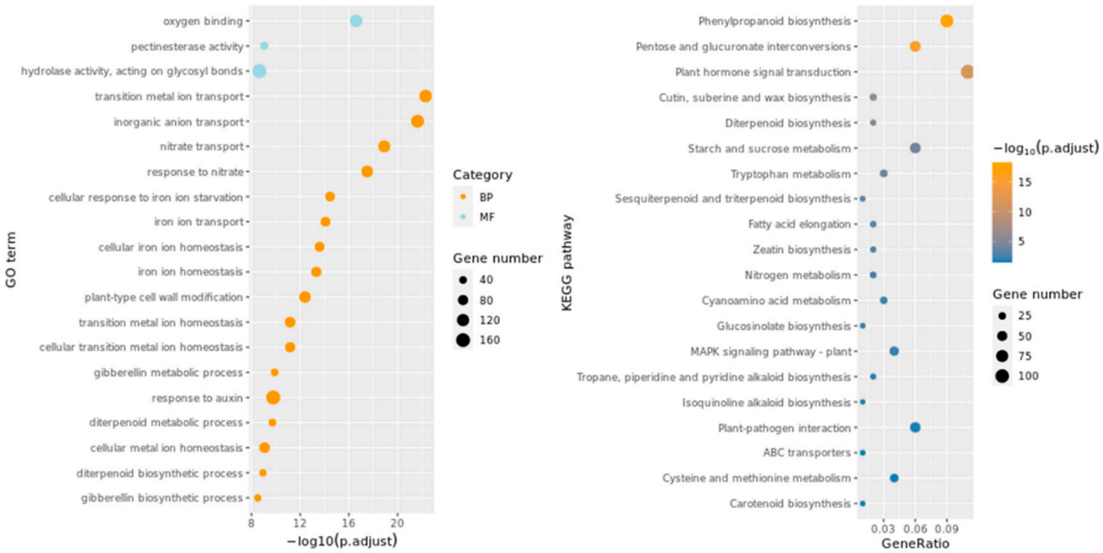

Figure S4. GO and KEGG pathway enrichment analysis of genes associated with histone modification enriched regions in Col-0.

Table S1. Primers used in this study.

Gene Symbol	Gene ID	Gene Description		Primers（5'-3'）
ACT2	AT3G18780	actin 2	F	CTTGCACCAAGCAGCATGAA
			R	CCGATCCAGACACTGTACTTCCTT
CPR5	AT5G64930	Constitutive expresser of PR genes 5	F	AGAGATTATGGACAACGACGAA
			R	CCACTCTCGTAACCCTTTTAGT
EGL3	AT1G63650	ENHANCER OF GLABRA 3	F	ATCAGCGAATTGTATGAACAGC
			R	TATCCGTTAGACCAGCATAACC
TTG1	AT5G245201	TRANSPARENT TESTA GLABRA 1	F	TTCCTTCGATTGGAACGATGTA
			R	GCAAGTCTTAACAAAGGCGTAT
GIS	AT3G58070	GLABROUS INFLORESCENCE STEMS	F	CAGTTAGGACTATGGCGTCTAC
			R	ATAGTTGAGTTTGAGAACGGGT
GL1	AT3G27920	GLABRA 1	F	ACTGGCTCAAAAAGATGTCCTA
			R	TAAGCTCAAAATCGTCGTCATG
GL3	AT5G41315	GLABRA 3	F	CAAAACGAGGAAGACGATTCAA
			R	CAACGCCTGAAGAAGAAGATTC
SAD	AT2G31660	SUPER SENSITIVE TO ABA AND DROUGHT2	F	TGGAGCTCTTTGTGACAAATTG
			R	GTGCAATGCTTTACGAAAGTTG
MYB23	AT5G40330	ATMYBRTF, MYB DOMAIN PROTEIN 23	F	AACACACATCTCAGCAAGAAAC
			R	GTATCGAACAGATTTGGAACCG
MYC1	AT4G00480	ATMYC1	F	CCATGTCCTATGTCTTCTCTCC
			R	GCTTCTTGCTAACAAAGAACGA
TT8	AT4G09820	TRANSPARENT TESTA 8	F	ACTAAAGATAAGAGGCTACCGC
			R	ATGATTTACGTACGCAATGGTG
CPC	AT2G46410	CAPRICE	F	TGAGTAGTATCGAATGGGAAGC
			R	GCCGTGTTTCATAAGCCAATAT
ETC1	AT1G01380	ENHANCER OF TRY AND CPC 1	F	AGTCGAAGCATCTTAAGACCAA
			R	ACCTTTCACCGACAAGCTTATA
ETC2	AT2G30420	ENHANCER OF TRY AND CPC 2	F	GCAAACTAAGTTCACTCGATCC
			R	CTCATTTGCCTTTCTTCCTACG
ETC3	AT4G01060	ENHANCER OF TRY AND CPC 3	F	ACCAACTCCATCGTTACTTCTT
			R	GCATTCGAGAGACCAAATCTTC
TCL1	AT2G30432	TRICHOMELESS1	F	CCAAGTTCACTCATAGCTCTCA
			R	CCCACCACTCTTCTTGCTATTA
TRY	AT5G53200	TRIPTYCHON	F	CGGTGATAGGTGGGATTTGATA
			R	TGGTATGTTTGTGGGAAGATGA

ZFP1	AT1G80730	ZINC-FINGER PROTEIN 1	F	TCTATAGTTCACAAGCGCTAGG
			R	CAAGGAGGAGAGAGTCATCTTG
ZFP5	AT1G10480	ZINC FINGER PROTEIN 5	F	TAACGACGTCATTTCAGAGACA
			R	GACCAATCTTCGTTGTACACAC
ZFP6	AT1G67030	ZINC FINGER PROTEIN 6	F	TTAAACGTGCACAGATGTTAGC
			R	AAGCCACTTAGAACTCGAAGAA
ZFP8	AT2G41940	ZINC FINGER PROTEIN 8	F	CGGAAGCTAGATCATACTACGG
			R	ACCTAAAGGACTTCCGTTGATT
IPT1	AT1G68460	isopentenyltransferase 1	F	GAATCGGAAAGACAAAGTCGTC
			R	ATGATCTCTGAAGGGAAACGAG
IPT3	AT3G63110	isopentenyltransferase 3	F	TTTGAGTTAGCTTGTAGGCAGA
			R	GACCTTCGCTTTGTAAAGACTG
IPT5	AT5G19040	isopentenyltransferase 5	F	GACACTTACGAAGATTTCACGG
			R	GCCTCGATGTAAGAATTGGAAC
CKX3	AT5G56970	cytokinin oxidase 3	F	GAACGAGTGATATCGAAAACGG
			R	TTCCTCGTTGACTGTGTATTGA
CKX4	AT4G29740	cytokinin oxidase 4	F	CCGGTTACATGGACGGATTATT
			R	TAATCTGAGGGCCGTGTCTAAA
CKX5	AT1G75450	cytokinin oxidase 5	F	GTATACACCGAGTTCGATGAGT
			R	GAGTTCGAGGCAATAAAGAACC
COI1	AT2G39940	CORONATINE INSENSITIVE 1	F	CAAGAGGTGTAAATTGAGCTGC
			R	CGTCTCGGAATCAATCTTGAAC
JAZ1	AT1G19180	JASMONATE-ZIM-DOMAIN PROTEIN 1	F	CGGGCAAGTGATTGTATTCAAT
			R	AGGAACTTGGTTTGCGATAGTA
